# Landmark models to define the age-adjusted risk of developing stage 1 type 1 diabetes across childhood and adolescence

**DOI:** 10.1186/s12916-019-1360-3

**Published:** 2019-07-09

**Authors:** Verena Sophia Hoffmann, Andreas Weiß, Christiane Winkler, Annette Knopff, Manja Jolink, Ezio Bonifacio, Anette-G. Ziegler

**Affiliations:** 1 Institute of Diabetes Research, Helmholtz Zentrum München, German Research Center for Environmental Health, Ingolstaedter Landstr. 1, 85764 Munich-Neuherberg, Germany; 2 Forschergruppe Diabetes e.V. at Helmholtz Zentrum München, German Research Center for Environmental Health, Munich-Neuherberg, Germany; 3 Technische Universität Dresden, DFG Center for Regenerative Therapies Dresden, Fetscherstrasse 105, 01307 Dresden, Germany; 4 Paul Langerhans Institute Dresden of the Helmholtz Center Munich at University Hospital Carl Gustav Carus and Faculty of Medicine, Dresden, TU Germany; 5 Forschergruppe Diabetes, Technical University Munich at Klinikum rechts der Isar, Munich, Germany

**Keywords:** Autoimmunity, Islet autoantibodies, Type 1 diabetes, Landmark risk

## Abstract

**Background:**

Autoimmune diseases are often preceded by an asymptomatic autoantibody-positive phase. In type 1 diabetes, the detection of autoantibodies to pancreatic islet antigens in genetically at-risk children is prognostic for future clinical diabetes. Testing for islet autoantibodies is, therefore, performed in a range of clinical studies. Accurate risk estimates that consider the a priori genetic risk and other risk modifiers are an important component of screening. The age of an individual is an under-appreciated risk modifier. The aim of this study was to provide age-adjusted risk estimates for the development of autoantibodies across childhood in genetically at-risk children.

**Methods:**

The prospective BABYDIAB and BABYDIET studies included 2441 children from birth who had a first-degree relative with type 1 diabetes. Children were born between 1989 and 2006 and were regularly followed from birth for the development of islet autoantibodies and diabetes. A landmark analysis was performed to estimate the risk of islet autoantibodies at birth and at the age 3.5, 6.5 and 12.5 years. Exponential decay curves were fitted for the risk by the age of 20 years.

**Results:**

The risk of islet autoantibodies by the age of 20 years was 8%, 4.6%, 2.6% and 0.9%, at the landmark ages of birth, 3.5, 6.5 and 12.5 years, respectively. The short-term risks (within 6 years of follow-up) at these landmark ages were 5.3%, 2.9%, 1.8% and 1%, respectively. The decline in autoantibody risk with age was modelled using a one-phase exponential decay curve (*r* = 0.99) with a risk half-life of 3.7 years. This risk decay model was remarkably consistent when the outcome was defined as islet autoantibody-positive or multiple islet autoantibody-positive and when the study cohort was stratified by HLA risk genotype. A similar decay model was observed for coeliac disease-associated transglutaminase antibodies in the same cohort. Unlike the risk of developing islet autoantibodies, the rate of developing clinical diabetes in children who were islet autoantibody-positive did not decline with age.

**Conclusion:**

The risk of developing autoantibodies drops exponentially with age in children with a first-degree relative with type 1 diabetes.

**Electronic supplementary material:**

The online version of this article (10.1186/s12916-019-1360-3) contains supplementary material, which is available to authorized users.

## Background

The early asymptomatic phase of type 1 diabetes is detected by the presence of autoantibodies against pancreatic β cell antigens such as insulin, glutamic acid decarboxylase (GAD), insulinoma-associated protein 2 (IA-2) and zinc transporter 8 (ZnT8) [1, 2]. Screening for islet autoantibodies is performed in people with a genetic predisposition for type 1 diabetes to investigate the natural history of the disease and when enrolling patients into interventional trials aimed at delaying the requirement for insulin replacement therapy [1, 3–9]. More recently, islet autoantibody screening has been piloted in the general population in Germany as part of routine healthcare to identify asymptomatic diabetes, known as stage 1 type 1 diabetes [10], and prevent diabetic ketoacidosis at the population level [11, 12].

An important aspect of offering tests for asymptomatic disease is the ability to determine accurate risk estimates for the group offered screening and to predict the prognosis of individuals with positive or negative test results. In cases where the a priori risk is increased, such as first-degree relatives of people with type 1 diabetes, it is important to provide them with accurate information on when to undergo testing and when their risk has changed. The risk of disease can change substantially with age. Although islet autoantibodies can appear throughout childhood and adolescence, they often appear early in life with a peak incidence period prior to 3 years of age [13–15]. Therefore, the risk of developing islet autoantibodies is likely to decline after this peak period of seroconversion. However, islet autoantibody risk estimates are rarely adjusted for the individual’s age. The aim of this study was to define the age-adjusted risk of developing stage 1 type 1 diabetes across childhood and adolescence. Risks were determined in first-degree relatives of patients with type 1 diabetes who participated in two long-running German birth cohort studies, which have prospectively followed individuals from birth, with nearly 30 years of follow-up.

## Methods

### Study population

We used data from two German birth cohorts of individuals with a first-degree family history of type 1 diabetes born between 1989 and 2006 [7, 8]. The studies prospectively examined the natural history of islet autoimmunity and type 1 diabetes. The BABYDIAB study enrolled 1650 infants born to a mother or father with type 1 diabetes, and the BABYDIET study enrolled 791 infants who had a mother, father, or sibling with type 1 diabetes. A subgroup of 150 children with high-risk human leukocyte antigen (HLA) genotypes or two or more first-degree relatives with type 1 diabetes participated in the BABYDIET gluten intervention study (ClinicalTrials.gov NCT01115621) to investigate whether delaying exposure to gluten could reduce the risk of developing autoantibodies. The intervention failed to show an effect on islet autoantibody development and all participants continued with follow-up examinations according to the natural history protocol [8]. Children enrolled in the BABYDIAB or BABYDIET studies were scheduled for follow-up and venous blood collection at 9 months and 2 years of age, and every 3 years thereafter, whereas the 150 children participating in the dietary intervention were followed up with venous blood collection every 3 months until 3 years of age and yearly thereafter.

### Assessments of islet autoantibodies and diabetes

Autoantibodies to insulin, GAD, IA-2 and ZnT8 were measured in samples taken at all scheduled visits and every 6 months in islet autoantibody-positive children. Transglutaminase autoantibodies were measured at all scheduled visits [16]. All autoantibody measurements were performed centrally by the Institute of Diabetes Research Munich using radiobinding assays and thresholds based on the upper 99th centile and Q-Q plots of results from control children as previously described [16–18]. Assay performance in international workshops is summarised in Additional file 1: Table S1.

Children were classified as islet autoantibody positive if they were positive for at least one islet autoantibody in at least two consecutive samples. The age of the first autoantibody-positive sample was considered the seroconversion age. Children were classified as multiple islet autoantibody-positive if, in addition to persisting islet autoantibody positivity, they tested positive for more than one islet autoantibody on at least one occasion. Genetic typing at HLA-*DRB1*, HLA-*DQA1* and HLA-*DQB1* loci was performed as previously described [19].

Oral glucose tolerance tests were performed annually in islet autoantibody-positive children. Type 1 diabetes was diagnosed according to the American Diabetes Association Expert Committee criteria [20]. Families of children who dropped out of the study or refused to provide blood samples or perform oral glucose tolerance tests were regularly contacted by telephone and were asked if the child had developed diabetes.

### Statistical analyses

To assess the time-dependent influence of age, cumulative islet autoantibody risks were calculated for children who remained islet autoantibody negative from birth or the respective landmark to the first islet autoantibody-positive state and to first multiple autoantibody-positive state. Cumulative risks were also calculated for progression to clinical diabetes in islet autoantibody-positive children. We determined landmark models [21] based on the cumulative incidence curves for the time from birth or from the respective landmark age to the first islet autoantibody-positive state, to the first multiple autoantibody-positive state and to clinical diabetes. The landmark ages were birth, 3.5, 6.5 and 12.5 years and were chosen at time points between scheduled visits to allow confirmation of positives prior to the landmark age. The follow-up time was set to 6 years (short-term risk), 12 years (mid-term risk) and 20 years (long-term risk).

Six-year dynamic prediction models were determined for all ages [22]. The prediction curves were produced using a Loess-based method [23]. Continuity correction, which accounted for the number at risk at each time point, was used to determine the 95% confidence intervals (CI) as previously described [24].

Single-phase exponential decay functions were generated on the risk by age 20 years and by 6 years and 12 years of follow-up from the landmark ages of birth, 1.5, 3.5, 6.5, 9.5 and 12.5 years to produce risk decay curves and equations and to calculate the risk half-life for each curve using the exponential decay curve fit function in GraphPad Prism. The function was constricted to a plateau risk of > 0%. The root mean square error was calculated as a measure of prediction error.

All analyses were performed separately for children with the HLA-DR3/4-DQ8 or HLA-DR4/4-DQ8 genotypes. Risks are given with 95% CIs in parentheses. Analyses were performed using R version 3.4.4 also using the package *dynpred* and GraphPad Prism version 8.0.1. (GraphPad Software, San Diego, CA, USA).

## Results

Overall, 2441 children (1188 girls; 49%) were enrolled at birth into the BABYDIAB and BABYDIET studies for prospective follow-up and were included in the present analyses. At the time of the analysis (December 2018), 164 children had developed at least one islet autoantibody, 132 had developed multiple islet autoantibodies and 115 had developed type 1 diabetes, including 10 children where type 1 diabetes was diagnosed without prior detection of islet autoantibodies.

### Landmark risks for islet autoantibodies

**Fig. 1:**
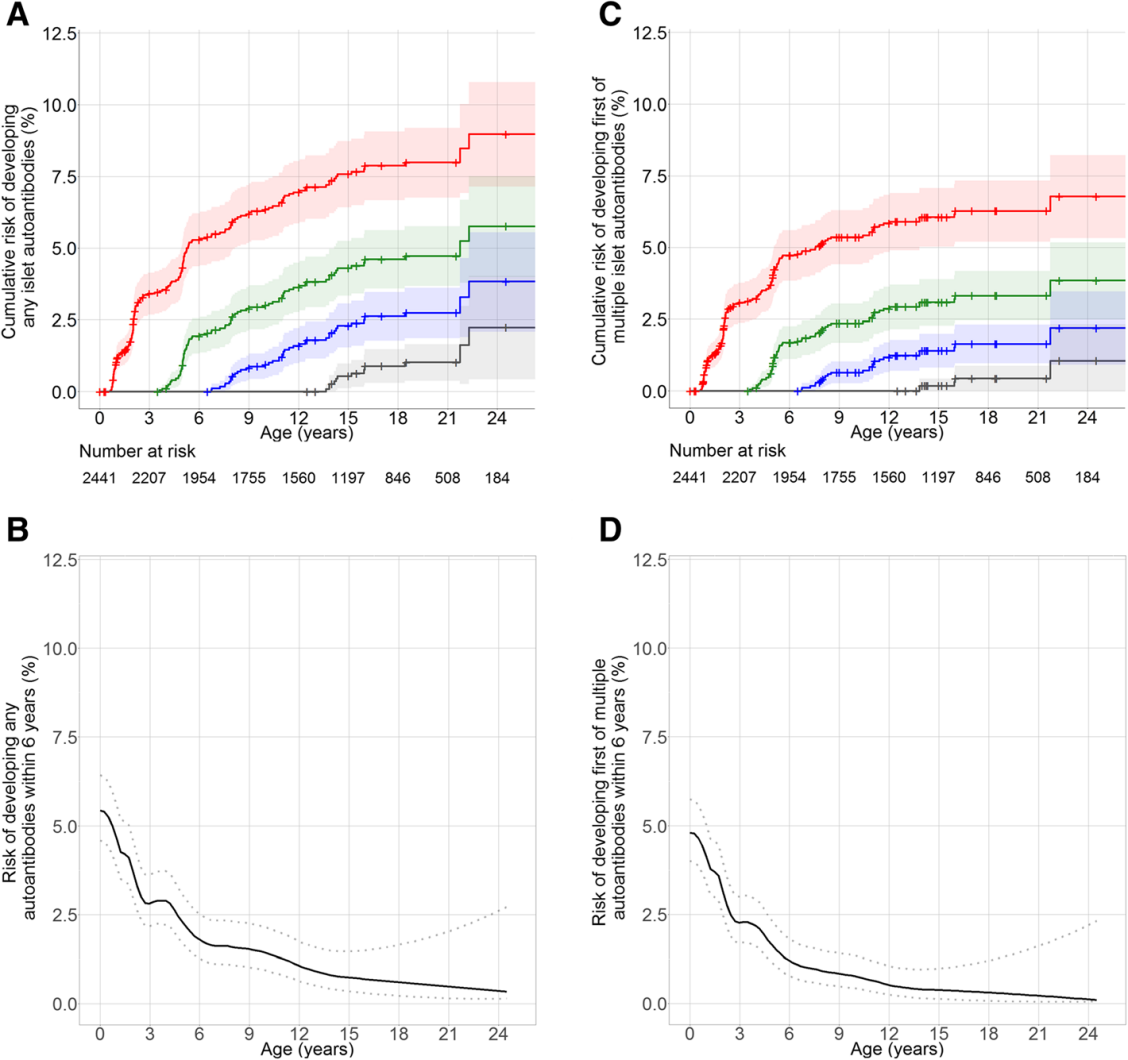
Cumulative risks of developing islet autoantibodies. **a**, **c** Cumulative risks of developing any (**a**) or multiple islet autoantibodies (**c**) in the total cohort from birth (red), 3.5 years (green), 6.5 years (blue) and 12.5 years (grey) of age. **b**, **d** Dynamic predictions for the risks of developing any (**b**) or multiple (**d**) islet autoantibodies during the next 6 years of life

Landmark models demonstrated decreasing short-term (6 years of follow-up), mid-term (12 years of follow-up) and long-term (20 years) risks of developing islet autoantibodies and multiple islet autoantibodies with increasing age of the children (Fig. 1, Table 1). The cumulative risks (95% CI) of developing islet autoantibodies after 12 years of follow-up from the landmark ages of birth, 3.5, 6.5 and 12.5 years were 7.0% (5.9–8.0%), 4.4% (3.4–5.3%), 2.7% (1.9–3.6%) and 2.2% (0.4–4.0%), respectively (Fig. 1a); and the 12-year cumulative risks (95% CI) for developing multiple islet autoantibodies were 5.8% (4.8–6.8%), 3.1% (2.3–3.9%), 1.6% (1.0–2.3%) and 1.1% (0–2.3%), respectively (Fig. 1b; Table 1). The short-term (6 years) risk of developing islet autoantibodies decreased rapidly with increasing age (Fig. 1b, d).

**Table 1:** Landmark model of cumulative risks of developing islet autoantibodies and type 1 diabetes

Outcome	Landmark age	Risk by 20 years of age	Risk after 6 years of follow-up	Risk after 12 years of follow-up
Any autoantibody	From birth	8.0% (6.8–9.2%)	5.3% (4.4–6.2%)	7.0% (5.9–8.0%)
	From 1.5 years	6.7% (5.5–7.8%)	4.2% (3.3–5.0%)	5.8% (4.8–6.9%)
	From 3.5 years	4.7% (3.7–5.8%)	2.9% (2.2–3.7%)	4.4% (3.4–5.3%)
	From 6.5 years	2.7% (1.9–3.6%)	1.8% (1.1–2.4%)	2.7% (1.9–3.6%)
	From 9.5 years	2.0% (1.2–2.8%)	1.6% (0.9–2.3%)	2.0% (1.2–2.8%)
	From 12.5 years	1.0% (0.4–1.7%)	1.0% (0.4–1.7%)	2.2% (0.4–4.0%)
Multiple autoantibodies	From birth	6.3% (5.2–7.3%)	4.7% (3.8–5.6%)	5.8% (4.8–6.8%)
	From 1.5 years	4.8% (3.9–5.8%)	3.7% (2.9–4.4%)	4.5% (3.6–5.4%)
	From 3.5 years	3.3% (2.5–4.2%)	2.4% (1.7–3.0%)	3.1% (2.3–3.9%)
	From 6.5 years	1.6% (1.0–2.3%)	1.2% (0.7–1.8%)	1.6% (1.0–2.3%)
	From 9.5 years	0.9% (0.4–1.4%)	0.7% (0.2–1.1%)	0.9% (0.4–1.4%)
	From 12.5 years	0.4% (0–0.9%)	0.4% (0–0.9%)	1.1% (0–2.3%)
Type 1 diabetes	From birth	6.2% (5.0–7.3%)	1.4% (0.9–1.9%)	3.7% (2.9–4.5%)
	From 1.5 years	5.9% (4.8–7.1%)	1.6% (1.1–2.2%)	4.2% (3.3–5.0%)
	From 3.5 years	5.4% (4.3–6.5%)	1.8% (1.2–2.3%)	4.2% (3.3–5.1%)
	From 6.5 years	4.7% (3.7–5.8%)	2.4% (1.7–3.1%)	4.5% (3.5–5.5%)
	From 9.5 years	3.7% (2.7–4.7%)	2.5% (1.7–3.2%)	3.7% (2.7–4.7%)
	From 12.5 years	2.4% (1.5–3.3%)	2.1% (1.3–2.9%)	2.8% (1.6–4.0%)
Type 1 diabetes, if islet antibody positive	From 1.5 years	83.6% (56.4–93.8%)	45.6% (22.7–61.7%)	54.3% (30.0–70.2%)
	From 3.5 years	84.3% (69.7–91.9%)	44.4% (30.4–55.6%)	70.7% (56.2–80.4%)
	From 6.5 years	76.2% (62.1–85.0%)	44.0% (32.2–53.8%)	71.3% (57.8–80.5%)
	From 9.5 years	64.1% (47.9–75.3%)	42.2% (29.0–53.0%)	64.1% (47.9–75.3%)
	From 12.5 years	47.8% (31.3–60.4%)	42.0% (26.8–54.1%)	47.8% (31.3–60.4%)

Cumulative risks (95% confidence intervals) of developing any islet autoantibody, the first of multiple islet autoantibodies, type 1 diabetes and type 1 diabetes if at least one autoantibody developed by the age of 20 years, and after 6 and 12 years of follow-up from the respective landmark age

**Fig. 2:**
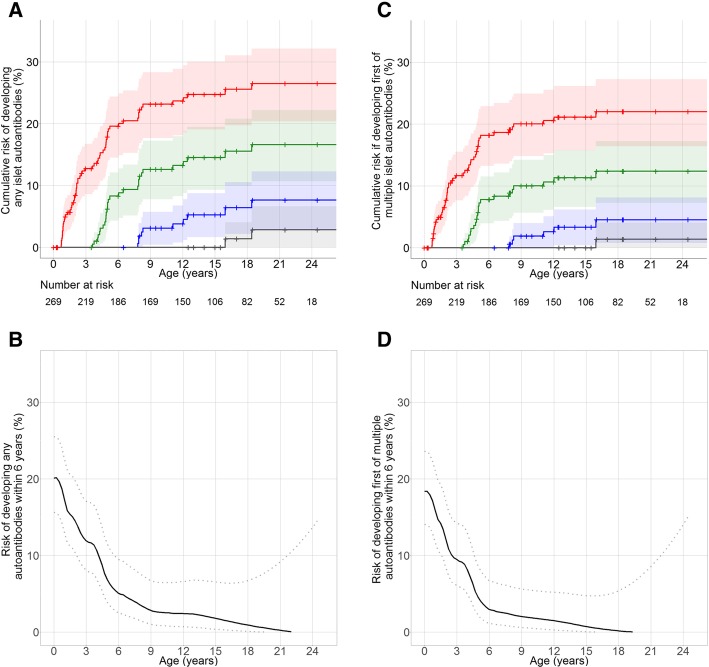
Cumulative risks of developing islet autoantibodies in children with DR3/4-DQ8 or DR4-DQ8/DR4-DQ8 genotypes. **a**, **c** Cumulative risks of developing any (**a**) or multiple islet autoantibodies (**c**) in children with DR3/4-DQ8 or DR4-DQ8/DR4-DQ8 genotypes from birth (red), 3.5 years (green), 6.5 years (blue) and 12.5 years (grey) of age. **b**, **d** Dynamic predictions for the risks of developing any (**b**) or multiple (**d**) islet autoantibodies during the next 6 years of life

Although risks were higher, similar relationships between landmark age and the risk of developing islet autoantibodies were observed when the analysis was restricted to children with the high-risk HLA-DR3-DR4-DQ8 or DR4-DQ8/DR4-DQ8 genotypes (Fig. 2, Additional file 1: Table S2).

### Modelling the age-related autoantibody risk decline

**Fig. 3:**
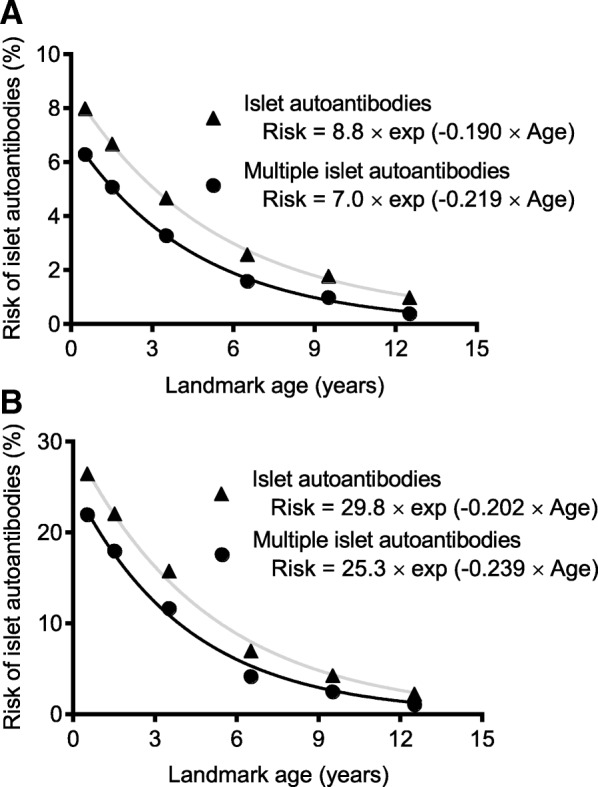
One-phase exponential decay function for risk of developing islet autoantibodies by 20 years of age. **a** All children. **b** Children with the high-risk HLA DR3-DR4-DQ8 or DR4-DQ8/DR4-DQ8 genotypes

The decline in risk of islet autoantibodies with increasing age was remarkably consistent regardless of the outcome and duration of follow-up. Therefore, we sought to model the rate of decline using a curve fit function. A one-phase exponential decay function described the risk reduction with landmark age (RMSE = 0.155 for any islet autoantibodies and 0.074 for multiple islet autoantibodies; Fig. 3a; Additional file 1: Figure S1). The risk half-life was 3.7 years (95% CI, 2.9–5.1 years) for developing any islet autoantibodies and 3.2 years (95% CI, 2.6–4.0 years) for developing multiple islet autoantibodies by the age of 20 years. The risk of developing islet autoantibodies by the age of 20 years for any given age was described by the equation: risk at age *X* = 8.8 × exp.(− 0.190 × age *X*), where age *X* is the age at testing in the first-degree relative. The equation for multiple islet autoantibodies was a risk at age *X* = 7 × exp.(− 0.219 × age *X*). For relatives with the high-risk HLA-DR3-DR4-DQ8 or DR4-DQ8/DR4-DQ8 genotypes, the exponential decay function yielded a risk half-life of 3.4 years (95% CI, 2.3–7.0 years) for any islet autoantibody and 2.9 years (95% CI, 2.2–4.2 years) for multiple islet autoantibodies by the age of 20 years (Fig. 3b).

Transglutaminase autoantibodies, which are associated with coeliac disease, were also measured in this cohort and provided us with an opportunity to test whether autoimmunity that often presents in childhood and with a partially known aetiology (exposure to gluten) displays a similar risk decay to islet autoimmunity (Additional file 1: Figure S2, Additional file 1: Table S3). The risk declined after the age of 1.5 years and was described by an exponential decay function risk at age *X* = 9.1 × exp.(− 0.290 × age *X*)) with a half-life of 4.2 years (95% CI, 3.2–6.1 years). An exponential decay function was also observed for children with the genotype DR3/3, which conveys high transglutaminase autoantibody risk (Additional file 1: Figure S2c).

### Landmark risks for progression from islet autoantibody positivity to clinical type 1 diabetes

**Fig. 4:**
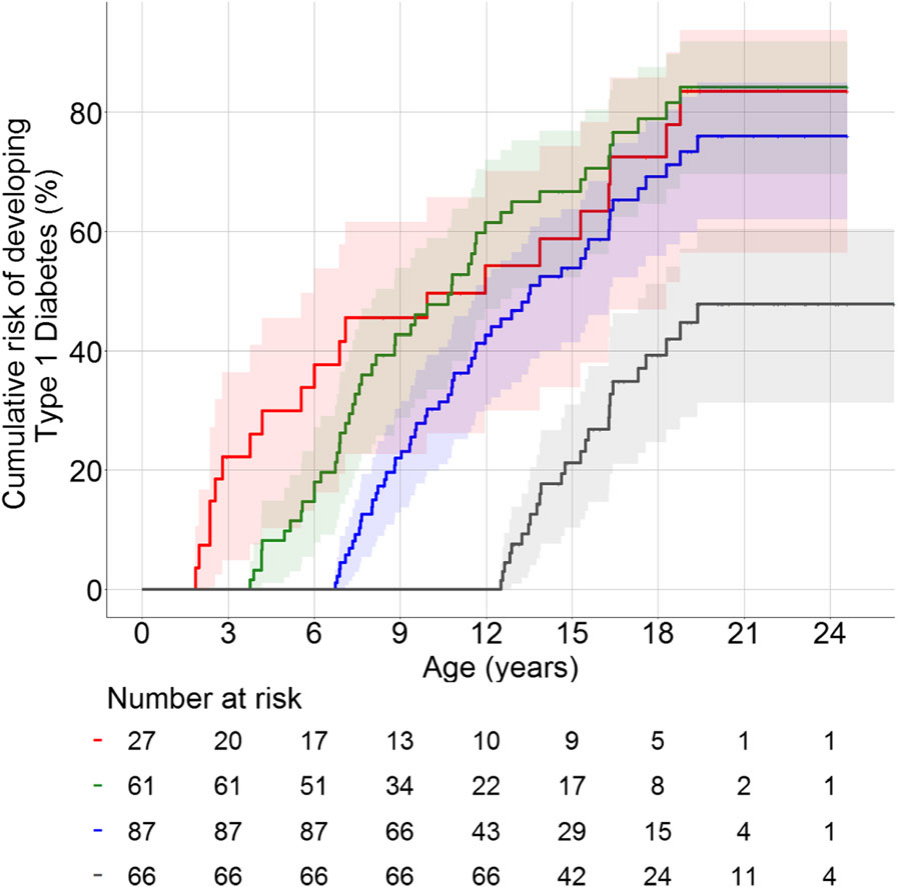
Cumulative risks of developing type 1 diabetes in islet autoantibody positive children. The cumulative risks were calculated from 1.5 years (red), 3.5 years (green), 6.5 years (blue), and 12.5 years (grey) in children who were islet autoantibody positive (multiple or single) at the respective landmark

Unlike the risk of developing islet autoantibodies, the 6-year and 12-year risks of developing clinical diabetes in islet autoantibody-positive children (Fig. 4) and in multiple islet autoantibody-positive children (Additional file 1: Figure S3) did not change with increasing age. The cumulative risks (95% CI) at 12 years of follow-up were 54.3% (30.0–70.2%), 70.7% (56.2–80.4%), 71.3% (57.8–80.5%) and 47.8% (31.3–60.4%) in children who tested positive for islet autoantibodies at the ages of 1.5, 3.5, 6.5 and 12.5 years, respectively.

## Discussion

In this study, we defined the influence of age on the risk of stage 1 type 1 diabetes among children with a first-degree family history of type 1 diabetes. The risks decreased exponentially with a risk half-life of between 2.9 and 3.7 years for islet autoantibodies depending upon the a priori genetic risk and whether the outcome was the development of any islet autoantibody or multiple islet autoantibodies. However, the prognosis for clinical diabetes in children who had developed islet autoantibodies did not change with increasing age.

This is the first demonstration of a mathematical function describing the risk of autoimmunity at landmark ages. The relationship was consistently described by a single-phase exponential decay function for short-, mid- and long-term risks. These functions differed in terms of the overall risk from birth, but not the risk half-life. The description of risk throughout childhood was possible because of the uniquely long follow-up of this cohort. It is also possible that a single-phase decay function was observed because of the relatively homogeneous nature of the cohort, which comprised children who had a first-degree relative with type 1 diabetes and who were followed from birth. It is known that the risk of developing islet autoantibodies differs according to which of the first-degree relatives is the proband [25, 26] and it is possible that the risk decay and the half-life may vary between children whose mother has type 1 diabetes and children whose father or sibling has type 1 diabetes. By restricting the analysis to children from affected families, we cannot assume that the risk decay is similar in children with non-familial genetic risk of type 1 diabetes. We and others have shown that autoimmunity to insulin often precedes autoimmunity to GAD [13–15], and the TEDDY study defined insulin-first and GAD-first endotypes [25]. It is, therefore, possible that the risk decay varies between each endotype and that the inclusion of other demographic and clinical factors, such as the child’s sex and family history status, may be used to refine the risk decay models.

The findings of the study have practical value. Parents from affected families are often concerned about their child’s risk of type 1 diabetes, which is 10–20 times higher than that of a child in an unaffected family. Screening for islet autoantibodies can alleviate the concern if the result is negative, but a common question is whether a negative result implies that the child is no longer at high risk. Our findings indicate that the risk is reduced by 4 times by the time the children start school and by nearly 16 times once the children reach their teens, bringing it to a level similar to that of infants in the general population. Based on our findings, we suggest that screening of islet autoantibodies in children from affected families may be most beneficial if performed at the age of 2–3 years, which is shortly after the peak incidence of islet autoantibodies [13–15], again at ~ 6 years of age or entrance to elementary school, and in the early teens, after which time the risk of developing islet autoantibodies is not zero but no longer markedly elevated. The findings are also relevant to defining optimal screening ages for cohort and intervention studies.

It is possible to speculate on what the exponential risk decay implies for disease pathogenesis. While genetics markedly stratifies the magnitude of the risk of developing islet autoimmunity, the exponential decay was consistent in the overall cohort and the high-risk HLA subgroup. A single-phase exponential decay curve can be explained by a single initiation period and random development of islet autoantibodies thereafter. This scenario may imply that many of the genetic and non-genetic factors that determine islet autoimmunity act in the earliest years of life. We sought to provide further evidence for this possibility by using coeliac disease-associated autoimmunity where dietary gluten is known to be a major non-genetic aetiological risk factor. The findings for the development of transglutaminase autoantibodies mirrored those of islet autoimmunity with a later start and a longer half-life. As already discussed, a caveat is that the discriminatory power of multiple different risk decay curves is low in this cohort, and it is possible that there are different sets of risk factors for different endotypes.

The lack of a risk decay for prognosis in children who had developed islet autoantibodies also has practical value. This implies that the risk of becoming insulin dependent for an islet autoantibody-positive child remains constant, regardless of the child’s age. Previous findings indicate that progression is faster in children who develop islet autoantibodies before the age of 3 years [1] and that progression is slower in autoantibody-positive adults than in autoantibody-positive children [27, 28]. Therefore, the prognosis may be defined by a complex function with more than one phase that was not discernible in our cohort. A two-phase decay may explain the substantial proportion of patients developing the clinical disease in adulthood without a requirement for a second wave of islet autoimmunity at older ages. Pathogenetically, the relatively long progression half-life is consistent with scenarios of random development of diabetes after autoimmunity, or scenarios with one or few influencing factors that may occur at any age or multiple influencing factors occurring at different ages.

## Conclusion

The development of islet autoimmunity in childhood follows an exponential decay model in affected families starting in the first year of life and with a risk half-life of 3–4 years.

## Additional file

Additional file 1: **Table S1.** Performance of the autoantibody assays used in the BABYDIAB and BABYDIET studies in the international Diabetes Autoantibody Standardisation Programme (DASP) and the Islet Autoantibodies Standardisation Programme (IASP). **Table S2.** Landmark model of cumulative risks of developing islet autoantibodies and type 1 diabetes in children with the DR3/4-DQ8 or DR4-DQ8/DR4-DQ8 genotypes. **Table S3.** Landmark model of cumulative risks of developing transglutaminase autoantibodies. **Figure S1.** One-phase exponential decay functions of single and multiple islet autoantibodies for 6-year (black) and 12-year follow-up (blue). The 6-year exponential decay functions are 5.9 × exp.(− 0.267 × age) and 5.3 × exp.(− 0.241 × age) for single and multiple autoantibodies respectively. The 12-year exponential decay functions are 7.8 × exp.(− 0.254 × age) and 6.6 × exp.(− 0.272 × age) for single and multiple autoantibodies respectively. **Figure S2.** Cumulative Risks of developing transglutaminase autoantibodies (total population (A) and children with DR3/3 genotype (B)) from birth (red), from 3.5 years of age (green), from 6.5 years (blue) and from 12.5 years (grey), and one-phase exponential decay curves for total cohort and the high risk HLA DR3-DR4-DQ8 or DR4-DQ8/DR4-DQ8 genotypes (C). **Figure S3.** Cumulative risks of developing type 1 diabetes in multiple islet autoantibody positive children. The cumulative risks were calculated from 1.5 years (red), 3.5 years (green), 6.5 years (blue) and 12.5 years (grey) in children who were multiple islet autoantibody positive at the respective landmark. (DOC 441 kb)

## Data Availability

The dataset analysed in this paper is available from the corresponding author on reasonable request, and with appropriate additional ethical approvals, where necessary.

## Abbreviations

CI: Confidence interval
GAD: Glutamic acid decarboxylase
HLA: Human leukocyte antigen
IA-2: Insulinoma-associated protein 2
ZnT8: Zinc transporter 8
