# Supplementary material for: Landmark models to define the age-adjusted risk of developing stage 1 type 1 diabetes across childhood and adolescence
Source: BMC Med. 2019 Jul 9;17:125. doi: 10.1186/s12916-019-1360-3 (PMC6615150; doi:10.1186/s12916-019-1360-3)
Supplement: Supplementary file 1 — Table S1. Performance of the autoantibody assays used in the BABYDIAB and BABYDIET studies in the international Diabetes Autoantibody Standardisation Programme (DASP) and the Islet Autoantibodies Standardisation Programme (IASP). Table S2. Landmark model of cumulative risks of developing islet autoantibodies and type 1 diabetes in children with the DR3/4-DQ8 or DR4-DQ8/DR4-DQ8 genotypes. Table S3. Landmark model of cumulative risks of developing transglutaminase autoantibodies. Figure S1. One-phase exponential decay functions of single and multiple islet autoantibodies for 6-year (black) and 12-year follow-up (blue). The 6-year exponential decay functions are 5.9 × exp.(− 0.267 × age) and 5.3 × exp.(− 0.241 × age) for single and multiple autoantibodies respectively. The 12-year exponential decay functions are 7.8 × exp.(− 0.254 × age) and 6.6 × exp.(− 0.272 × age) for single and multiple autoantibodies respectively. Figure S2. Cumulative Risks of developing transglutaminase autoantibodies (total population (A) and children with DR3/3 genotype (B)) from birth (red), from 3.5 years of age (green), from 6.5 years (blue) and from 12.5 years (grey), and one-phase exponential decay curves for total cohort and the high risk HLA DR3-DR4-DQ8 or DR4-DQ8/DR4-DQ8 genotypes (C). Figure S3. Cumulative risks of developing type 1 diabetes in multiple islet autoantibody positive children. The cumulative risks were calculated from 1.5 years (red), 3.5 years (green), 6.5 years (blue) and 12.5 years (grey) in children who were multiple islet autoantibody positive at the respective landmark. (DOC 441 kb) [file 12916_2019_1360_MOESM1_ESM.doc]

**Landmark models to define the age-adjusted risk of developing Stage 1 type 1 diabetes across childhood and adolescence**

Verena Sophia Hoffmann^1^, Andreas Weiß^1^, Christiane Winkler^1,2^, Annette Knopff^1^, Manja Jolink^1,2^, Ezio Bonifacio^2,3,4+^, Anette-G. Ziegler^1,2,5+^

**Additional File 1**

**Additional File Table 1**

Performance of the autoantibody assays used in the BABYDIAB and BABYDIET studies in the international Diabetes Autoantibody Standardization Programme (DASP) and the Islet Autoantibodies Standardization Programme (IASP).

| **Year** | **IAA** | | | **GADA** | | | **IA-2A** | | | **ZnT8A*** | | |  |
| --- | --- | --- | --- | --- | --- | --- | --- | --- | --- | --- | --- | --- | --- |
|  | AUC | Sens  % | Spec  % | AUC | Sens  % | Spec  % | AUC | Sens  % | Spec  % |  | Sens  % | Spec  % | |
| 2000 | 0.75 | 30 | 98 | 0.95 | 80 | 94 | 0.74 | 58 | 100 |  |  |  | |
| 2002 | 0.81 | 50 | 98 | 0.96 | 86 | 94 | 0.76 | 62 | 100 |  |  |  | |
| 2003 | 0.85 | 64 | 99 | 0.95 | 84 | 96 | 0.84 | 66 | 100 |  |  |  | |
| 2005 | 0.87 | 70 | 99 | 0.94 | 86 | 93 | 0.86 | 72 | 100 |  |  |  | |
| 2007 | 0.90 | 78 | 97 | 0.98 | 92 | 100 | 0.84 | 71 | 100 |  |  |  | |
| 2009 | 0.85 | 66 | 99 | 0.94 | 68 | 94 | 0.88 | 60 | 99 |  | 72 | 99 | |
| 2010 | 0.75 | 42 | 100 | 0.93 | 84 | 92 | 0.86 | 66 | 99 |  | 70 | 99 | |
| 2012 | 0.79 | 48 | 99 | 0.84 | 64 | 93 | 0.88 | 72 | 100 |  | 66 | 99 | |
| 2013 | 0.73 | 40 | 99 | 0.90 | 67 | 99 | 0.84 | 66 | 100 |  | 68 | 98 | |
| 2015 | 0.83 | 70 | 98 | 0.89 | 76 | 99 | 0.90 | 74 | 100 |  | 74 | 100 | |
| 2016 | 0.79 | 54 | 99 | 0.93 | 66 | 99 | 0.90 | 76 | 100 |  | 64 | 99 | |

*ZnT8 autoantibodies were not included in the programmes until 2009

** Area Under the Curve

DASP and IASP included 150 coded sera (100 adult blood donors; and 50 patients with recent onset type 1 diabetes) that were sent to multiple laboratories every 18 months. Although many of these were used in multiple send outs, no send out contained exactly the same sera. In particular the patient sera that were used to calculate sensitivity varied considerably between send outs and variation in the sensitivity between send outs is expected. Three parameters were provided to participants: the AUC, which is the area under the curve of the ROC plot analysis of the laboratory data, the sensitivity, which is the proportion of the patient sample called positive and the specificity, which is the proportion of the blood donor samples called negative. The AUC could not be determined for ZnT8 autoantibodies due to two separate assays used to determine positivity. For GAD autoantibodies, the blood donor samples included some samples that were consistently positive in radiobinding assays and which were no longer included in recent send outs.

**Additional File Table 2**

Landmark model of cumulative risks of developing islet autoantibodies and type 1 diabetes in children with the DR3/4-DQ8 or DR4-DQ8/DR4-DQ8 genotypes

| **Outcome** | **Landmark age** | **Risk by 20 years of age** | **Risk after 6 years of follow-up** | **Risk after 12 years of follow-up** |
| --- | --- | --- | --- | --- |
| Any autoantibody | From birth | 26.5% (20.4%–32.2%) | 19.6% (14.5%–24.4%) | 23.7% (18.1%–28.9%) |
|  | From 1.5 years | 22.2% (16.1%–27.8%) | 15.7% (10.9%–20.4%) | 20.3% (14.8%–25.5%) |
|  | From 3.5 years | 16.6% (10.7%–22.2%) | 12.6% (7.8%–17.2%) | 14.5% (9.3%–19.5%) |
|  | From 6.5 years | 7.6% (2.8%–12.3%) | 5.3% (1.6%–8.8%) | 7.6% (2.8%–12.3%) |
|  | From 9.5 years | 5.1% (0.5%–9.5%) | 2.4% (0%–5.1%) | 5.1% (0.5%–9.5%) |
|  | From 12.5 years | 2.8% (0%–6.6%) | 2.8% (0%–6.6%) | 2.8% (0%–6.6%) |
| Multiple autoantibodies | From birth | 22.0% (16.4%–27.3%) | 18.2% (13.2%–22.9%) | 20.6% (15.3%–25.5%) |
|  | From 1.5 years | 18.0% (12.6%–23.1%) | 14.5% (9.8%–18.9%) | 17.1% (12.0%–21.9%) |
|  | From 3.5 years | 12.4% (7.3%–17.2%) | 10.0% (5.6%–14.2%) | 11.3% (6.6%–15.8%) |
|  | From 6.5 years | 4.5% (0.8%–8.1%) | 3.3% (0.4%–6.2%) | 4.5% (0.8%–8.1%) |
|  | From 9.5 years | 2.9% (0%–6.2%) | 1.6% (0%–3.8%) | 2.9% (0%–6.2%) |
|  | From 12.5 years | 1.4% (0%–4.1%) | 1.4% (0%–4.1%) | 1.4% (0%–4.1%) |
| Type 1 diabetes | From birth | 20.8% (15.0%–26.3%) | 5.4% (2.6%–8.1%) | 13.6% (9.2%–17.7%) |
|  | From 1.5 years | 19.9% (14.1%–25.3%) | 5.9% (3.0%–8.7%) | 13.5% (9.9%–18.9%) |
|  | From 3.5 years | 19.0% (13.3%–24.4%) | 7.7% (4.3%–11.0%) | 15.3% (10.4%–19.9%) |
|  | From 6.5 years | 16.0% (10.4%–21.2%) | 9.3% (5.4%–13.0%) | 16.0% (10.4%–21.2%) |
|  | From 9.5 years | 12.3% (7.0%–17.2%) | 8.2% (4.2%–12.1%) | 12.3% (7.0%–17.2%) |
|  | From 12.5 years | 7.4% (2.8%–11.9%) | 7.4% (2.8%–11.9%) | 7.4% (2.8%–11.9%) |

Cumulative risks (95% confidence intervals) of developing any islet autoantibody, the first of multiple islet autoantibodies or type 1 diabetes by the age of 20 years, and after 6 and 12 years of follow-up from the respective landmark age in children with a DR3/4-DQ8 or DR4-DQ8/DR4-DQ8 genotype.

**Additional File Table 3**

Landmark model of cumulative risks of developing transglutaminase autoantibodies

| **Study population** | **Landmark age** | **Risk at 20 years of age** | **Risk after 6 years of follow-up** | **Risk after 12 years of follow-up** |
| --- | --- | --- | --- | --- |
| Total cohort | From birth | 6.3% (5.2%–7.4%) | 3.3% (2.6%–4.1%) | 5.5% (4.5%–6.5%) |
|  | From 1.5 years | 6.3% (5.1%–7.4%) | 3.8% (3.0%–4.7%) | 5.6% (4.5%–6.6%) |
|  | From 3.5 years | 4.6% (3.6%–5.7%) | 3.1% (2.3%–3.9%) | 4.3% (3.3%–5.2%) |
|  | From 6.5 years | 2.9% (2.0%–3.7%) | 2.1% (1.4%–2.8%) | 2.9% (2.0%–3.7%) |
|  | From 9.5 years | 1.7% (0.9%–2.4%) | 1.2% (0.6%–1.8%) | 1.7% (0.9%–2.4%) |
|  | From 12.5 years | 0.8% (0.2%–1.3%) | 0.8% (0.2%–1.3%) | 0.8% (0.2%–1.3%) |
| HLA DR3/3 children | From birth | 36.0% (20.9%–48.2%) | 19.0% (8.7%–28.2%) | 29.9% (17.2%–40.6%) |
|  | From 1.5 years | 36.0% (20.9%–48.2%) | 22.6% (11.4%–32.4%) | 29.9% (17.2%–40.6%) |
|  | From 3.5 years | 30.7% (15.4%–43.3%) | 20.2% (8.7%–30.2%) | 26.6% (13.5%–37.8%) |
|  | From 6.5 years | 20.9% (5.9%–33.5%) | 13.4% (2.8%–22.8%) | 20.9% (5.9%–33.5%) |
|  | From 9.5 years | 13.2% (0%–25.2%) | 8.1% (0%–16.6%) | 13.2% (0%–25.2%) |
|  | From 12.5 years | 8.7% (0%–19.9%) | 8.7% (0%–19.9%) | 8.7% (0%–19.9%) |

Cumulative risks (95% confidence intervals) of developing transglutaminase autoantibodies by the age of 20 years, and after 6 and 12 years of follow-up from the respective landmark age in the total cohort and in children with the DR3/3 genotype.

**Additional File Figure 1**

One-phase exponential decay functions of single and multiple islet autoantibodies for 6 year (black) and 12 year follow-up (blue). The 6 year exponential decay functions are 5.9 × exp(-0.267 × age) and 5.3 × exp(-0.241 × age) for single and multiple autoantibodies respectively. The 12 year exponential decay functions are 7.8 × exp(-0.254 × age) and 6.6 × exp(-0.272 × age) for single and multiple autoantibodies respectively.

**Additional File Figure 2**

**C** **C**

Cumulative Risks of developing transglutaminase autoantibodies (total population (A) and children with DR3/3 genotype (B)) from birth (red), from 3.5 years of age (green), from 6.5 years (blue) and from 12.5 years (grey), and one-phase exponential decay curves for total cohort and the high risk HLA DR3-DR4-DQ8 or DR4-DQ8/DR4-DQ8 genotypes (C).

**Additional File Figure 3**

Cumulative risks of developing type 1 diabetes in multiple islet autoantibody positive children. The cumulative risks were calculated from 1.5 years (red), 3.5 years (green), 6.5 years (blue), and 12.5 years (grey) in children who were multiple islet autoantibody positive at the respective landmark.
